# Impact of Helmet Use in Traumatic Brain Injuries Associated with Recreational Vehicles

**DOI:** 10.1155/2013/450195

**Published:** 2013-09-25

**Authors:** Latha Ganti, Aakash N. Bodhit, Yasamin Daneshvar, Pratik Shashikant Patel, Christa Pulvino, Kelsey Hatchitt, Robyn M. Hoelle, Keith R. Peters, Sudeep Kuchibhotla, Lawrence Lottenberg, Andrea Gabrielli, Anna Mazzuoccolo, Marie-Carmelle Elie-Turenne, Tricia Falgiani, Porter W. Maerz, Shivam M. Kharod, Lauren M. Conroy, Hussain M. Khalid, J. Adrian Tyndall

**Affiliations:** ^1^Center for Brain Injury Research and Education, University of Florida College of Medicine, 1329 SW 16th Street, Suite 4270, Gainesville, FL 32610, USA; ^2^Department of Emergency Medicine, University of Florida College of Medicine, Gainesville, FL 32610, USA; ^3^Department of Neurological Surgery, University of Florida College of Medicine, Gainesville, FL 32610, USA; ^4^Division of Clinical Research, Emergency Medicine & Neurological Surgery, Toral Family Foundation, Davie, FL 33325, USA; ^5^Department of Radiology, University of Florida College of Medicine, Gainesville, FL 32610, USA; ^6^Department of Anesthesiology, University of Florida College of Medicine, Gainesville, FL 32610, USA

## Abstract

*Objective*. To study the impact of helmet use on outcomes after recreational vehicle accidents. *Methods*. This is an observational cohort of adult and pediatric patients who sustained a TBI while riding a recreational vehicle. Recreational vehicles included bicycles, motorcycles, and all-terrain vehicles (ATVs), as well as a category for other vehicles such as skateboards and scooters. *Results*. Lack of helmet use was significantly associated with having a more severe traumatic brain injury and being admitted to the hospital. Similarly, 25% of those who did wearing a helmet were admitted to the ICU versus 36% of those who did not (*P* = 0.0489). The hospital length of stay was significantly greater for patients who did not use helmets. *Conclusion*. Lack of helmet use is significantly correlated with abnormal neuroimaging and admission to the hospital and ICU; these data support a call for action to implement more widespread injury prevention and helmet safety education and advocacy.

## 1. Introduction

In recent years, the use of recreational vehicles (RVs), such as on- and off-road motorcycles and all-terrain vehicles (ATVs), has increased significantly in popularity. Nearly one in five Americans (19.2%) aged sixteen and older participated one or more times in off-highway recreation within the past year. The use of these vehicles is especially popular in the under-thirty age group [1]. Unfortunately, RV use puts a person at risk of sustaining a traumatic brain injury (TBI), which is a leading cause of injury-related death and disability in the US [2]. Incidence of these injuries as a result of RV use may be on the rise. While the overall rate of motor vehicle-related TBI deaths decreased between 1993 and 2007, the rate of motorcycle-related TBI deaths actually increased significantly during those years [2]. In fact, although motorcycles account for only 2% of vehicle registrations in the US, motorcycle accidents are responsible for 10% of traffic-related deaths [3]. Bicycle riding is also a common activity particularly among children. In USA, approximately 70% of children aged from 5 to 14 ride bicycles. Head and brain injuries during a crash are the worst danger associated with bicycle riding. According to the US Centers for Disease Control, head injury is the most common cause of death and serious disability from bicycle crashes.

In addition to being a leading cause of death, TBIs can dramatically diminish quality of life for patients who survive. In one study, employment rate prior to sustaining a moderate or severe TBI was 80%; at three months after injury, employment rate was 15%, and at 3 years after injury, employment rate had only increased to 55% [4]. Furthermore, the high incidence of TBIs in the US presents a major public health problem since patients with these injuries often end up seeking medical care later, sometimes long after the injury, with complaints of ongoing symptoms [5]. 

Therefore, preventative measures must be implemented to reverse the trend of recreational vehicle-related TBIs. The first step is to document the burden of the problem. In this study we sought to study the impact of helmet use on the clinical outcomes after head injury.

## 2. Methods

This IRB approved study is an observational cohort of adult and pediatric patients who presented to the emergency department following a TBI while riding a recreational vehicle during a 30-month period from January 2008 to August 2010. Full methodological details have been previously reported [6]. Briefly, cohort identification was accomplished using the following ICD-9 codes: 800.0−801.9, 803.0−804.9, 850.0−854.1, 995.55, 959.01, and 950.1−950.3. The injury e code matrix was used to classify vehicles as “on-road” or “off-road” [7]. The study was conducted at a level-1 trauma center located in a college town of 50,000 students with a 13-county catchment area in the North Central Florida region where the recreational use of off-road vehicles is particularly high. Recreational vehicles included bicycles, motorcycles, and all-terrain vehicles (ATVs), as well as a category for other vehicles such as skateboards and scooters. Data were collected for 482 patients. It was a subset of a cohort that included all traumatic brain injury patients regardless of their mechanism of injury. Data included demographic information (age, gender, race, etc.); prehospital care data when available (prehospital GCS score); and information about their injury (mechanism of injury, signs and symptoms associated with injury). Signs, and symptoms included loss of consciousness (LOC), alteration of consciousness (AOC), posttraumatic amnesia (PTA), any episode of vomiting after injury, and any reported seizure activity after injury. Outcome variables included ED admission TBI severity, clinical data, head CT findings, need for hospital admission, and ICU admission. Data were also collected for 72-hour return to ED and 30-day hospital readmission.

 Data were entered into research electronic data capture (REDCap), which is a secure, web-based application hosted by our institution's center for translational science institute (CTSI) and is designed to support traditional clinical data capture. Statistical analyses were performed in JMP 9.0 (SAS institute, Cary, NC, USA) for the Macintosh.

## 3. Results

The cohort of 478 was 75% males. The median age was 25 years (IQR 17–44, range 2–85). There were 143 (30%) bicycle riders, 168 (35%) motorcycle riders, and 91 (19%) ATV riders, and 66 (14%) were on skateboards, scooters, or other recreational vehicles; 10 (2%) were involved in watercraft accidents (Table 1). Tables 2 and 3 summarize the numbers of patients who came in as transfers versus directly from the scene, with the corresponding number of deaths in each category.  Table 3 further breaks down this information by TBI severity and again delineates the number of deaths.  Table 4 provides age ranges for each vehicles type.

Helmet use ranged from 14 to 46% in different vehicle types (Table 5). Helmet was use was a significant predictor of TBI severity, when controlling for vehicle type and gender of patient. For analytical purpose, we combined patients with GCS scores of 3–8 and 9–12 in one group, as we had few patients with GCS score of 9–12. Helmet use was associated with less severe TBI (OR = 2.1, 95% CI = 1.19–3.81). TBI severity was significantly worse in both the prehospital (*P* = 0.015) and ED (*P* = 0.012) environments when a helmet was not worn (Table 6). Helmet use was associated with significantly less likelihood of having an abnormal CT (*P* = 0.0085, OR = 0.56, CI = 0.36–0.86). Of the CT abnormalities (Table 7), 92% were bleeds and 61.5% were fractures. Any abnormal bleeding (included epidural, subdural, subarachnoid, intraparenchymal, and any contusion) on head CT scan was more significantly more common in nonhelmeted patients compared to helmeted patients (*P* = 0.043, OR = 1.58, CI = 1.01–2.48). This remains true for the finding of fracture (skull fracture including calvarial fractures through carotid canal and foramen magnum) on head CT scan (*P* = 0.0005, OR = 2.53, CI = 1.48–4.33). Overall, 11% required neurosurgical intervention (ventriculostomy or craniotomy) for their injury.

Lack of helmet use was associated with higher hospital admission rate (59.5% versus 50.7%), but it was not statistically significant. Similarly, 25% of those wearing a helmet were admitted to the ICU, whereas this number rose to 35% for those not wearing a helmet (*P* = 0.04). The maximum length of stay in the hospital was significantly greater for patients who did not use helmets (144 days versus 46 days for helmet users). Thirty-day readmission rate was also higher for nonhelmeted patients (6%) than for helmeted patients (2%).

Alcohol use before injury was significantly higher among those not wearing a helmet compared to those who did (23% versus 2%, *P* < 0.0001). Also, 66.7% of those without helmet had a positive drug screen (28 out of 42 screened) compared to 50% (9 out of 18) for those with helmets. Urine drug screening can detect cannabinoid, amphetamines, cocaine, barbiturates, and phencyclidine.

Injury clinical presentations by helmet use are summarized in Table 8. There were not any statistically significant differences in signs and symptoms after TBI in patients wearing helmet compared to those without helmets.

## 4. Discussion

These data demonstrate that lack of helmet use is significantly correlated with abnormal head CT scans, admission to the ICU, and worse TBI severity. Also, helmet use is associated with less hospital admission rate. This relationship between helmet use and TBI severity has been supported by other studies done to show the preventive ability of helmets against the effects of impact on human skull models. Studies have shown that bicycle helmet use reduces the risk of brain injury by 88% [8]. A recent study that conducted crush tests using human cadaver skulls demonstrated that wearing a helmet can reduce the acceleration experienced by up to 87% during impact and also help the skull in resisting forces up to 47 pounds [9]. Population-based studies conducted to find out effectiveness of helmets in reducing severity of head injuries have reported results similar to those found in our study. In a review of the literature regarding motorcycle helmet use, “the authors found voluminous support for motorcycle helmet use as a way to prevent severe TBI and traffic fatalities” [3]. In a review conducted by Thompson et al., the authors reported that helmets reduce bicycle-related head and face injuries in bicyclists of all ages regardless of crash type [10]. A recent case-control study conducted in Canada reported a significant increase in mortality associated with nonhelmet use in bicyclists compared to helmet use [11]. Similarly, the effectiveness of helmet use in reducing risk of head injury and mortality in motorcycle riders is well documented [12, 13]. Preventive effects of helmets were also reported in moped or scooter riders in a study published by Hooten and Murad [14]. A West Virginia study [15] found that nonhelmeted riders were significantly more likely to be admitted to the hospital and sustain more injuries. A similar epidemiological review of pediatric ATV riders in Tennessee [16] notes that helmet use resulted in fewer injuries to the head, neck, and face. These findings demonstrate the benefit of wearing helmets during recreational vehicle use and suggest a call for action to implement more widespread injury prevention and helmet safety education and advocacy. 

 Implementation of universal laws for helmet use has been a controversial issue. The arguments against the universal helmet use laws are reduction of freedom, perceived inconvenience to use helmets, and even more chances of cervical injuries in helmet users after certain speed due to mechanism involved [17, 18]. The concerns about increased chances of cervical spine injuries in helmet users have been addressed in other studies, and it has been reported that helmet use does not increase chances of cervical spine injuries [19, 20], but in fact it can reduce such injuries [21]. In an Italian study conducted to compare the impact of compulsory helmet use, it was found that, after adoption of law, there was significant decrease in neurosurgical hospital admissions for motorcycle- or moped-related TBI. This decrease in TBIs was evident in all age groups. One significant finding was that epidural hematomas were rare after the adoption of law, which was enforced strictly by police [22]. Currently, only 20 states and District of Columbia have helmet laws that apply to each age group. A study that used the cross-sectional time series data from National Highway Traffic Safety Administration (NHTSA) for a period of 1975–2004 demonstrated that states with universal helmet laws have approximately 22–33% lower fatality rates for motorcycle users compared to states without universal helmet laws. Same study found that partial coverage helmet laws also reduce fatality in motorcycle riders by about 7–10% [23]. Dao et al. reported that hospital admitted patients in states with mandatory helmet laws have decreased rate of cervical spine injuries compared to those in states with flexible helmet laws [24]. A statewide hunter education program in Arkansas for ATV safety has been reported to be successful in a survey-based study [25]. 

 Along with establishment and strict implementation of universal helmet laws, health education programs focused on children, adolescents, and young adults will most likely improve the helmet use rates. A large number of children, adolescents, and young adults use recreational vehicles, and a lot of them are inexperienced and are less likely to follow traffic rules [17]. Community participation should be an integral part of such programs as it improves effectiveness and success rates of such programs. Community-based and collaborative approach is necessary for such programs to succeed, as only education can increase the safety knowledge in targeted population, but not necessarily the safe behavior by targeted population [26].

Study limitations included the following. Our data were significant in an aspect that they included all recreational vehicle types; however, the actual type and quality, proper fitting, and mechanics of the helmets were not studied. Furthermore, no significant details on the kinetics of the impact were available, such as speed and angle of impact and body mass of subjects. Indeed, one study on hockey helmets found that the current methods of safety testing may miss some important risks, and additional testing conditions should be added to existing test protocols [27]. Therefore, the best way to decrease the occurrence of RV-related TBIs is to provide public education about the relationship between these injuries, recreational vehicles, and helmet use while simultaneously conducting research to design more effective helmets and enforce their safety standard. Also, there were relatively few moderate TBIs in our cohort. The reason for this may be that a significant percentage of TBIs that are initially moderate go on to become severe very quickly, either because of intrinsic injury/pathology or because of how GCS gets reported. 

## 5. Conclusion

These data demonstrate that lack of helmet use is significantly correlated with abnormal head CT scans (fractures), admission to the hospital, admission to the ICU, and overall worse TBI severity both in the prehospital and ED environments. These results underscore the importance of wearing helmets during recreational vehicle use and suggest a similar benefit for all recreational activity at high risk of brain impact. We join others in supporting a call for action to implement more widespread injury prevention and helmet safety education and advocacy.

## 6. What Is Already Known on This Subject

- Helmet use is a good preventative measure against head injury.
- Many states have helmet laws, especially for children; unfortunately not all states do.

## 7. What This Study Adds

- Lack of helmet use is significantly correlated with numerous concrete outcome measures including neurological deficits, radiological signs of skull fracture, and admission to the hospital and ICU.
- Overall, not wearing a helmet resulted in worse TBI severity both in the prehospital and ED environments.
- Presenting these updated statistics is important especially in light of the rise in recreational vehicle use.

## Figures and Tables

**Table 1:** Classification of vehicles as on- or off-road, per injury e code matrix.

Type of vehicle/animal (other)	On-road	Off-road
Scooter (*n* = 28)	22	6
Skateboard (*n* = 18)	0	18
Golf cart (*n* = 11)	0	11
Horse (*n* = 10)	0	10
Bicycle (*n* = 143)	93	50
Motorcycle (*n* = 167)	127	40
ATV (*n* = 91)	3	88

**Table 2:** Types of vehicles, transfer status, and deaths.

Type of vehicle	Transfer	No transfer	Deaths
Bicycle (*n* = 143)	18	125	5 (1,4)
Motorcycle (*n* = 167)	21	146	15 (1,14)
ATV (*n* = 91)	20	71	1 (1,0)
Other (*n* = 67)	7	60	2 (0,2)
Watercraft (*n* = 10)	4	6	0

Total (*n* = 478)	70	408	23 (3,20)

**Table 3:** Types of vehicles and presenting GCS.

Type of vehicle	GCS 13–15	GCS 9–12	GCS 3–8	Deaths
Bicycle (*n* = 143)	132 (92.3%)	2 (1.4%)	9 (6.3%)	5 (3.5%)
Motorcycle (*n* = 167)	111 (66.5%)	8 (4.8%)	48 (28.7%)	15 (9%)
ATV (*n* = 91)	69 (75.8%)	1 (1.1%)	21 (23.1%)	1 (1.1%)
Other (*n* = 67)	58 (86.6%)	4 (6%)	5 (7.4%)	2 (3%)
Scooter (*n* = 28)	23 (82.1%)	2 (7.2%)	3 (10.7%)	2 (7.1%)
Skateboard (*n* = 18)	18 (100%)	0	0	0
Golf cart (*n* = 11)	9 (81.8%)	2 (18.2%)	0	0
Horse (*n* = 10)	8 (80%)	0	2 (20%)	0
Watercraft (*n* = 10)	10 (100%)	0	0	0

Patients excluding transferred				
Bicycle (*n* = 125)	116 (92.8%)	0	9 (7.2%)	4 (3.2%)
Motorcycle (*n* = 146)	98 (67.1%)	7 (4.8%)	41 (28.1%)	14 (9.6%)
ATV (*n* = 71)	54 (76.1%)	1 (1.4%)	16 (22.5%)	0
Other (*n* = 60)	52 (86.7%)	3 (5%)	5 (8.3%)	2 (3%)
Scooter (*n* = 28)	23 (82.1%)	2 (7.2%)	3 (10.7%)	2 (7.1%)
Skateboard (*n* = 16)	16 (100%)	0	0	0
Golf cart (*n* = 8)	7 (87.5%)	1 (12.5%)	0	0
Horse (*n* = 8)	6 (75%)	0	2 (25%)	0
Watercraft (*n* = 6)	6 (100%)	0	0	0

**Table 4:** Vehicle type by age range.

	0–4 y	5–14 y	15–19 y	20–24 y	25–34 y	35–44 y	45–54 y	55–64 y	≥65 y
Whole cohort (*n* = 478)	10 (2.1%)	80 (16.7%)	75 (15.7%)	70 (14.6%)	71 (14.9%)	53 (11.1%)	65 (13.6%)	38 (8%)	16 (3.3%)
Bicycle (*n* = 143)	4 (2.8%)	20 (14%)	16 (11.2%)	27 (18.9%)	20 (14%)	14 (9.8%)	21 (14.7%)	16 (11.2%)	5 (3.5%)
Motorcycle (*n* = 167)	0	16 (9.6%)	22 (13.2%)	21 (12.5%)	25 (15%)	28 (16.7%)	30 (18%)	17 (10.2%)	8 (4.8%)
ATV (*n* = 91)	4 (4.4%)	28 (30.7%)	18 (19.8%)	7 (7.7%)	18 (19.8%)	7 (7.7%)	6 (6.6%)	1 (1.1%)	2 (2.2%)
Other (*n* = 67)	2 (3%)	15 (22.4%)	15 (22.4%)	13 (19.4%)	7 (10.4%)	3 (4.5%)	7 (10.4%)	4 (6%)	1 (1.5%)
Scooter (*n* = 28)	2 (7.1%)	5 (17.9%)	3 (10.7%)	9 (32.1%)	2 (7.1%)	1 (3.6%)	4 (14.3%)	2 (7.2%)	0
Skateboard (*n* = 16)	0	5 (27.8%)	9 (50%)	4 (22.2%)	0	0	0	0	0
Golf cart (*n* = 8)	0	2 (18.2%)	2 (18.2%)	0	4 (36.3%)	1 (9.1%)	1 (9.1%)	0	1 (9.1%)
Horse (*n* = 8)	0	3 (30%)	1 (10%)	0	1 (10%)	1 (10%)	1 (10%)	2 (20%)	0
Watercraft (*n* = 10)	0	1 (10%)	4 (40%)	2 (20%)	1 (10%)	1 (10%)	1 (10%)	0	0

**Table 5:** Characteristics of riders of different recreational vehicles.

	+ helmet	% males riding recreational vehicles	Median age (IQR; range)
Bicycle (*n* = 143)	29 (20%)	73	26 (19–48; 3–85)
Motorcycle (*n* = 168)	77 (46%)	81	33 (21–49; 6–70)
ATV (*n* = 91)	13 (14%)	70	18 (13–32; 2–72)
Other (skateboard, scooter) (*n* = 54)*	9 (14%)	70	19 (14–28; 4–58)

*12 patients were excluded as they were injured while using vehicles where normally the riders do not wear helmets such as a golf cart. Also watercraft injuries are not included in the table.

**Table 6:** TBI severity and symptoms at injury based on helmet use.

	+ helmet (*N* = 128)	No helmet (*N* = 328)
Mild (GCS 13–15)	105 (82%)	255 (77.7%)
Moderate (GCS 9–12)	2 (1.5%)	11 (3.3%)
Severe (GCS 3–8)	21 (16.4%)	62 (18.9%)

**Table 7:** CT abnormalities by helmet use.

CT abnormality (*n* = 420)	Helmeted (*n* = 121)	Nonhelmeted (*n* = 299)	*P* value
Cranial and intracranial			
Fracture of skull	13 (10.7%)	68 (22.7%)	0.004
Calvarial fracture through carotid canal/foramen magnum	0	8 (2.71)	—
Epidural, subdural, and subarachnoid hemorrhages	39 (32.2%)	133 (44.5%)	0.02
Intraparenchymal hemorrhage including intraventricular hemorrhage and contusions	22 (18.2%)	90 (30.1%)	0.01
Diffuse axonal injury	9 (7.4%)	13 (4.3%)	NS

Extracranial			
Fractures of maxillo-facial bones	14 (11.6%)	61 (20.4%)	0.033
Extracalvarial soft tissue swelling/defect	7 (5.8%)	66 (22%)	<0.0001

**Table 8:** Injury characteristics.

	+ helmet (*N* = 128)	No helmet (*N* = 328)	Not applicable (*N* = 22)
Associated vomiting	12 (9.4%)	28 (8.7%)	
Associated seizure	7 (5.5%)	14 (3%)	
LOC	98 (76%)	220 (67%)	15 (68.1%)
LOC > 30	16 (16.5%)	39 (18.5%)	
AOC	82 (64%)	210 (64%)	12 (54.5%)
PTA	55 (43%)	136 (41.5%)	8 (36.4%)

LOC: loss of consciousness; AOC: alteration in consciousness; PTA: posttraumatic amnesia.
